# Impact of CYP2C19*2 on Clopidogrel Response and Cardiovascular Outcomes in ST-segment elevation myocardial infarction Patients

**DOI:** 10.12669/pjms.42.1.12358

**Published:** 2026-01

**Authors:** Abdur Razaq, Waheed lqbal, Syed Tahir Shah, Mohsin Ali, Sami Siraj

**Affiliations:** 1 Abdur Razaq, Institute of Pharmaceutical Sciences, Khyber Medical University Peshawar, Pakistan; 2 Waheed lqbal, Institute of Pharmaceutical Sciences, Khyber Medical University Peshawar, Pakistan; 3 Syed Tahir Shah, Department of Cardiology, Kuwait Teaching Hospital, Peshawar, Pakistan; 4 Mohsin Ali, Laboratory of Pharmaceutical Biotechnology, Faculty of Pharmaceutical Sciences, Ghent University, Ghent, Belgium; 5 Sami Siraj, Institute of Pharmaceutical Sciences, Khyber Medical University Peshawar, Pakistan

**Keywords:** CYP2C19 *2 gene polymorphisms, Clopidogrel, Cardiovascular Events (CVEs) Percutaneous coronary intervention (PCI), ST-segment elevation myocardial infarction (STEMI)

## Abstract

**Background and Objective:**

Clopidogrel is essential to prevent cardiovascular events in patients undergoing primary percutaneous coronary intervention (PCI) for ST-segment elevation myocardial infarction (STEMI). Despite adherence to clopidogrel, a significant number of cardiovascular events (CVEs) occur in patients after angioplasty. In this study, we sought to determine the association of CVEs with genetic polymorphisms in CYP2C19*2 (rs4244285) that affect metabolic activation of clopidogrel.

**Methodology:**

A prospective cohort study (n=204) was conducted from August 2022 to March 2023 at Khyber Medical University and Kuwait Teaching Hospital in Peshawar, Pakistan. STEMI patients (age 30-75 years, all genders) undergoing PCI were included and followed for 12 months. Genotyping of CYP2C19*2 (rs4244285) was performed by TaqMan assay. CVEs (mortality, stent thrombosis, recurrent MI, ischemic events and stroke) were compared between wild-type and variant genotypes. Statistical analysis used Fisher’s exact test to compare CVEs between wild and mutant group, while binary logistic regression examined the relationship between CVEs and risk factors (SPSS v22).

**Results:**

The CYP2C19*2 (rs4244285) GA and AA genotypes were significantly associated with cardiovascular events (CVEs) (p < 0.003), whereas the wild-type GG genotype showed no significant correlation. During the 12-month follow-up after PCI, CVEs included: mortality (n = 10; GG = 2, GA+AA = 8), stent thrombosis (n = 5; GG = 0, GA+AA = 5), recurrent myocardial infarction (n = 9; GG = 1, GA+AA = 8), ischemia-related hospitalizations (n = 17; GG = 1, GA+AA = 16), and cerebrovascular accidents (n = 3; GG = 0, GA+AA = 3)

**Conclusion:**

Individuals carrying one or two non-functional CYP2C19*2 (rs4244285) alleles — GA and AA genotypes classified as intermediate and poor metabolizers, respectively— - showed a significant association with CVEs. Conversely, subjects with GG genotypes (normal metabolizers) had a significantly lower incidence of CVEs.

## INTRODUCTION

Dual antiplatelet therapy consisting of aspirin and clopidogrel is essential to prevent the cardiovascular events in patients undergoing primary percutaneous coronary intervention (PCI), for ST-segment elevation myocardial infarction (STEMI).^1^ Clopidogrel, a thienopyridine antiplatelet agent, requires hepatic biotransformation via the cytochrome P450 isoenzyme CYP2C19 to produce its active metabolite, which irreversibly binds to the P2Y12 adenosine diphosphate receptor on platelets, preventing platelet activation and aggregation.^2^ Studies have shown that CYP2C19 loss- of-function (LoF) allelic variants, particularly CYP2C19 *2 (rs4244285), significantly impair the bioactivation of clopidogrel, resulting in reduced platelet inhibition and increased risk of major adverse cardiovascular events (MACE) in STEMI patients undergoing PCI.^3^ Non-responsiveness to clopidogrel affects approximately 20-25% of patients undergoing PCI and poses an increased risk of adverse cardiovascular events.^4^ However, a wide range of inter-individual variability in clopidogrel’s antiplatelet efficacy has been demonstrated by numerous investigations.^5^ Clopidogrel is still widely prescribed in STEMI patients in the south Asia and other less developed areas in the world.^6^ Cardiovascular disease is highly prevalent in the Khyber Pakhtunkhwa province of Pakistan.^7^ The aim of this study was to determine the CYP2C19 *2 genotype and analyze the association between genotype and cardiovascular events as well as clinical characteristics and adverse cardiovascular outcomes during 12 months of follow-up in STEMI patients undergoing primary PCI. CYP2C19 *2 genotyping could improve treatment plans and advance personalized antiplatelet therapy in this population.

## METHODOLOGY

This was a prospective, single-center cohort study, conducted on 204 patients having first-time ST-elevation myocardial infarction (STEMI) undergoing PCI in the Kuwait Teaching Hospital, Peshawar, Pakistan between August 2022 and May 2024.

### Ethical approval:

This study was approved from Kuwait Teaching Hospital, Peshawar (Letter No. KTH 01/08-2022) and ethical review committee of the Institute of Pharmaceutical Sciences, Khyber Medical University, Peshawar, Pakistan (approval number: KMU/IPS/PG/IREB/2nd meeting/2024/6; Date: August 21, 2024).

### Inclusion criteria:

The inclusion criteria include persons aged 30-75 years of all genders who have been diagnosed with STEMI. This is defined as acute myocardial ischemia lasting >30 minutes with symptom onset <12 hours and electrocardiographic evidence of ST-segment elevation at the J-point ≥1 mm (0.1 mV) in at least two contiguous leads. Gender and lead specific criteria include: Males ≥40 years requiring ≥2 mm of elevation in leads V2-V3, males <40 years requiring ≥2.5 mm of elevation in leads V2-V3, and females of any age requiring ≥1.5 mm of elevation in leads V2-V3. In addition, a new or presumed new left bundle branch block (LBBB) with concomitant symptoms was considered STEMI-equivalent.^8^ All patients were treated according to the current American College of Cardiology/American Heart Association (ACC/AHA) and European Society of Cardiology (ESC) guidelines.^9^

### Exclusion criteria:

Patients with previous MI, patients that were using antiplatelet drug rather than Clopidogrel were excluded, patients with history of stroke, transient ischemic attack, platelets <100 000/μl, known bleeding disorder**,** severe liver problems, CKD patients with creatinine clearance less than 30ml/min and patient not consenting to participate in the study were also excluded.

### Data collection and follow-up:

A questionnaire as well as written consent was filled out of all patients. Demographic and anthropometric data were noted on day first. Those patients to whom drug eluting stent/stents Everolimus implanted; Peripheral blood samples (3-5 ml) were collected from each participant at the time of the procedure for subsequent genetic analysis. Extraction of genomic DNA from peripheral blood lymphocytes was performed using the salting out technique. DNA concentration and purity were assessed by spectrophotometric analysis using the NanoDrop™ 2000 platform (NanoDrop Technologies Inc., Wilmington, DE, USA). Complete blood count, Renal function test, Liver function test, Random blood sugar and Lipid profile was performed on day of enrollment. Patients had received 600mg loading dose of clopidogrel following by a dose of 75 mg once daily. Assigned treatment strategy was followed. There was follow up of all patients for duration of one month, three months and one year. The following clinical outcomes were registered: Death, stent thrombosis, recurrent MI, Ischemic events that need hospitalization and stroke. Death was defined as due to cardiovascular causes or any death without another known cause**.**

### TaqMan Genotyping assay for CYP2C19 *2 (rs4244285):

The TaqMan genotyping was performed at Ghent university, Belgium where 10-50 ng/μl of extracted genomic DNA was used with Applied Biosystems™ TaqPath™ ProAmp™ Master Mix (order number: 1140277922, catalog number: 15622139), which contains Taq DNA polymerase, dNTPs, buffers and other important components for the reaction and the TaqMan™ Drug Metabolism Genotyping Assay (20x) contains sequence-specific forward and reverse primers and two probes labeled with different fluorophores (FAM and VIC) for detection, Assay ID: C__25986767_70, catalog number: 4362691, order number: 4205309283) was used. The thermal cycling protocol included a pre-read step at (60°C for 30 seconds, followed by an initial denaturation at 95°C for five minutes. This was succeeded by 40 cycles of denaturation at 95°C for 30 seconds, annealing at 60°C for 30 seconds, and extension at 60°C for 30 seconds, concluding with a final extension at 60°C for 30 seconds.

### Statistical analysis:

Categorical variables were expressed as absolute values and percentages, whereas continuous data were presented as means with their standard deviation. Genotype and alleles frequencies were determined to assess whether the variants alleles are in Hardy- Weinberg equilibrium (HWE) in KPK, Pakistani population. Fisher’s exact test was used for CVEs versus CYP2C19 *2 genotype while, binary logistic regression was used to find the cardiovascular events vs various risk variables. The p-value ≤ 0.05 was considered to be statistically significance.

## RESULTS

**Fig.1:**
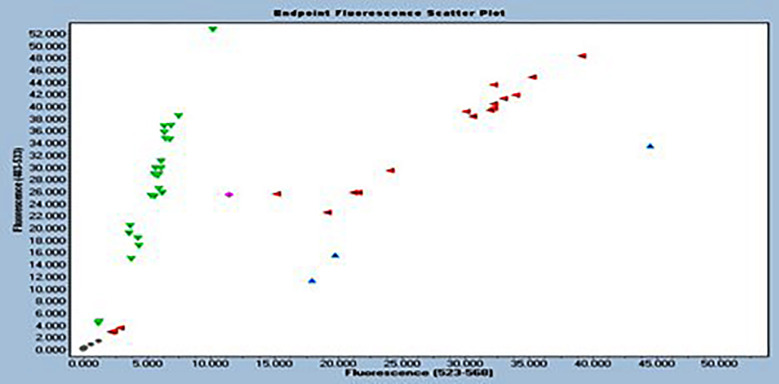
Allelic discrimination plot of CYP2C19*2 (rs4244285) by TaqMan genotyping. Fig.1 depicts the allelic discrimination analysis of CYP2C19*2 (rs4244285) by TaqMan genotyping. Wild-type G allele (FAM fluorescence, 483-533 nm) is plotted on the Y-axis, while mutant A allele (VIC fluorescence, 523-568 nm) is plotted on the X-axis. Negative controls appear at the plot origin while one mutant homozygous was used as positive control. Three distinct genotype clusters are evident: homozygous wild-type (GG) along the Y-axis, homozygous mutant (AA) along the X-axis, and heterozygous (GA) in the central region.

Out of 218 patients, follow-up was successful in 204 (93%). Loss to follow-up cases were 14 (7%). The mean follow-up duration was 375 **±** 15 days. Of the 204 STEMI patients, 122 (59.8%) were male, and 82 (40.2%) were female. The mean age was 59.90 ± 8.76 years. Demographics, blood investigations, and blood pressure are mentioned in Table-I. The study reports the results of the genotyping of CYP2C19 *2 in 204 individuals. The wild-type homozygous genotype (GG) was found in 86 individuals (42.15%), the heterozygous genotype (GA) in 116 individuals (56.86%) and the mutant homozygous genotype (AA) in two individuals (0,98%) (Table-II). The allele discrimination plot from TaqMan genotyping of CYP2C19*2 (rs4244285) for the wild-type G allele (FAM fluorescence, 483-533 nm) and the mutant A allele (VIC fluorescence, 523-568 nm) is shown in Fig.1.

**Table-I:** Descriptive Statistics

Demographic attributes	N	Minimum	Maximum	Mean	Std. Deviation
Age (years)	204	35.00	75.00	59.90	8.76
Weight (Kg)	204	44.00	140.00	73.58	15.54
Height (cm)	204	148.00	179.00	164.34	7.94
Body mass index	204	15.00	48.90	27.31	5.89
Hemoglobin (g/dl)	204	9.00	18.60	13.68	1.72
Platelets	204	151000.00	1145000.00	287068.2.	81758.71
White blood cells	204	3300.00	19200.00	8691.68	2532.13
Random blood sugar (mg/dl)	204	67.00	456.00	146.59	82.06
Serum creatinine (mg/dl)	204	0.40	2.10	0.96	0.26
Urea (mg/dl)	204	12.00	145.00	33.73	17.17
SGPT (U/L)	204	12.00	458.00	67.38	75.46
ALP (U/L)	204	23.00	321.00	105.16	60.10
Total cholesterol (mg/dl)	204	112.00	521.00	209.31	75.89
LDL (mg/dl)	204	41.00	175.00	94.72	32.08
HDL (mg/dl)	204	25.00	118.00	54.87	22.12
Triglyceride (mg/dl)	204	80.00	465.00	244.23	82.79
Troponin (ng/dl)	204	7.00	44467.00	3234.71	5087.41
Ejection fraction (%)	204	25.00	66.00	52.57	10.63
Systolic blood pressure (mmHg)	204	80.00	180.00	133.78	18.69
Diastolic blood pressure (mmHg)	204	55.00	120.00	84.75	11.30

**Table-II:** CYP2C19 *2 Genotyping and Allelic frequency

Genotype	Number of individual (%)	Frequency of G allele	Frequency of A allele
GG (Wild Homozygous)	86 (42.15%)	0.7059	0.2941
GA (Heterozygous)	116 (56.86%)		
AA (Mutant Homozygous)	2 (0.98%)		
Total	204 (100%)		

Association of CVEs with CYP2C19 *2 genotyping (wild type: GG and Mutant type: GA+AA) was found statistically significant (p-value < 0.003) (Table-III). The temporal distribution of cardiovascular events (CVEs) over a follow-up period of one year in 204 patients showed that a total of 44 cardiovascular events occurred (21.56% of patients), with the majority occurring in the first month after the procedure (21 events). The individual events listed in Table-IV.

**Table-III:** Association of CYP2C19 *2 genotyping (wild type: GG and Mutant type: GA+AA) with cardiovascular events using Fisher’s Exact Test

Outcomes	Genotype	Patients with Cardiovascular events		Total	p-value
		Yes	No		
Death	GG	2	84	86	0.19
	GA+AA	8	110	118	
Stent Thrombosis	GG	0	86	86	0.07
	GA+AA	5	113	118	
Recurrent MI	GG	1	85	86	0.08
	GA+AA	8	110	118	
Ischemic events	GG	1	85	86	0.004
	GA+AA	16	102	118	
Stroke	GG	0	86	86	0.26
	GA+AA	3	115	118	

**Table-IV:** Cardiovascular Events at different follow up (n=204)

Event Type	First Month	One month to Four Months	Four months to One Year	Total (%)
Death	4	4	2	10 (4.9%)
Stent Thrombosis	3	2	0	5 (2.45%)
Recurrent MI	4	3	2	9 (4.41%)
Ischemic Events	8	6	3	17(8.33%)
Stroke	2	1	0	3 (1.47%)
Total events	21	16	7	44 (21.56%)

The results of the binary logistic regression analyzing the associations between different risk factors and cardiovascular events in which CYP2C19 *2 mutant group (GA+ AA) had the highest odds ratio (3.076) and (p=0.003) which was statistically significant while age: patients aged 56-65 years had odds ratio (2.780) and (p=0.042) was also statistically significant. The number of diseased vessels was associated with CVEs compared to single-vessel disease (p=0.006), with (OR=2.487). The P-values for smoking and family history were (p=0.024) and (p=0.040), respectively, showing a strong association with CVEs (Table-V). These results underline the importance of age, extent of coronary disease, smoking status and family history as potential risk factors for adverse cardiovascular outcomes.

**Table-V:** Binary logistic regression for Cardiovascular events vs various risk variables

Variable	Types/category	Frequency	CVEs (Yes/No)	Odd Ratio	95% CI for Odd Ratio		p-value
					Lower	Upper	
CYP2C19 *2 Genotypes	GG	86	10/76	-			0.003
	GA+AA	118	34/84	3.07	1.42	6.64	
Gender	Female	82	15/67	-			0.35
	Male	122	29/93	0.71	0.35	1.44	
CAD patients based on age categories	Age ≤ 45 Years	10	2/8	-			0.042
	Age 46-55 Years	58	6/52	1.33	0.23	7.63	
	Age 56- 65 Years	69	21/48	2.78	0.92	8.38	
	Age 66-75 Years	67	15/52	0.59	0.25	1.39	
No of disease vessels	SVCAD	46	4/42	-			0.006
	DVCAD	64	22/42	2.48	0.79	7.83	
	TVCAD	94	18/76	0.45	0.21	0.93	
No of Stents used	One	104	18/86	-			0.177
	Two	66	20/46	0.27	0.09	0.85	
	Three	34	6/28	0.43	0.12	1.45	
Current smoker	No	188	37/151	-			0.024
	Yes	16	7/9	0.30	0.10	0.85	
Hypertension	No	101	21/80	-			0.673
	Yes	103	23/80	1.26	0.54	2.57	
Diabetes Mellitus	No	120	27/93	-			0.554
	Yes	88	17/71	1.26	0.58	2.70	
Dyslipidemia	No	185	39/146	-			0.717
	Yes	19	5/14	0.79	0.23	2.68	
Family history	No	117	19/98	-			0.040
	Yes	87	25/62	0.44	0.20	0.96	
Ejection fraction	40 greater	165	33/132	-	-	-	0.26
	40 less than	39	11/28	0.63	0.287	1.40	

## DISCUSSION

In our study, a high prevalence of CYP2C19 *2 (rs4244285) gene polymorphism was found in the Pakistani population on clopidogrel therapy, with 57.84% of patients carrying loss-of-function alleles. The 12 months cardiovascular event analysis demonstrated significant genotype-dependent outcome disparities, Using the TaqMan assay for genotype analysis, with GA+AA variant carriers exhibiting markedly elevated rates of adverse events compared to GG homozygotes across all measured parameters (p=0.003). The GA+AA group accounted for 80% of mortality events, all stent thromboses, 89% of recurrent myocardial infarctions, 94% of ischemia-related hospitalizations, and all cerebrovascular accidents, suggesting a strong association between genetic polymorphism and increased post-PCI cardiovascular risk. These findings indicate that variant carriers may exhibit altered drug metabolism and platelet function predisposing them to thrombotic complications despite standard antiplatelet therapy. The overall cardiovascular events during the one year follow-up period included ischemic events requiring hospitalization (8.33%), stent thrombosis (2.45%), recurrent myocardial infarction (4.41%), cerebrovascular events (1.47%) and cardiovascular death (4.9%). Multivariate logistic regression analysis identified several independent predictors of cardiovascular events, like CYP2C19 *2, odd ratio: 3.07, 95% confidence interval: 1.42-6.64 and p-value 0.003 and age (especially the age group 56-65 years, odds ratio: 2.780, 95% confidence interval: 0.922-8.381), extent of coronary artery involvement, family history and smoking status. Our finding of 57.84% prevalence of CYP2C19 *2 alleles with loss of function is in good agreement with previous Pakistani studies.^10^,^11^ across This concordance multiple Pakistani cohorts suggests a true population-specific genetic trait that is distinctly different from Western populations. A study conducted in Khyber Pakhtunkhwa revealed that intermediate metabolizers comprised 4% to 41% of the population, while poor metabolizers ranged from 0% to 20% across different ethnic groups in the province.^12^

Similarly, a retrospective cohort study of 160 patients with ST elevation myocardial infarction (STEMI) conducted in Karachi reported major adverse cardiac events (MACE) in 32.5% of patients, which is higher than our overall event rate.^13^ Our mortality rate of 4.9% is remarkably consistent with the J-PCI OUTCOME registry, which reported 6.8% mortality suggesting similar post-PCI outcomes despite different genetic backgrounds.^14^ A comprehensive meta-analysis found that adverse cardiovascular events occur in 10% to 32% of patients within 12 months after PCI, which is consistent with our results.^15^ The higher prevalence of loss-of-function alleles in Pakistani populations compared to Western cohorts can be attributed to distinct genetic ancestry and population-specific allele frequencies.^16^ The TaqMan assay provides an efficient and rapid method for the detection of CYP2C19 *2 variants and is a major advance in pharmacogenetic testing for personalized antiplatelet therapy in resource-limited clinical settings.^17^ The high prevalence of intermediate metabolizers observed in our study population suggests that alternative antiplatelet agents may be required for a substantial proportion of patients.

### Strength of the Study:

This finding suggests the potential benefit of alternative P2Y12 receptor antagonists such as ticagrelor or prasugrel in appropriately selected patients, as these agents are not affected by CYP2C19 polymorphisms.^18^ This study adds several important pieces of information and this is among the first comprehensive studies to establish CYP2C19 *2 allele frequencies specifically in Pakistani patients undergoing PCI, filling a critical gap in regional pharmacogenetic data. Our study uniquely combines genetic polymorphism data with traditional cardiovascular risk factors, demonstrating that genetic testing enhances risk stratification beyond conventional clinical parameters. This study has several methodological strengths, including its prospective design, comprehensive genetic analysis and rigorous clinical follow-up protocols.

### Limitations

Several limitations must be acknowledged, including the exclusion of other CYP2C19 polymorphisms especially (*3, *4, and *17), the single-center design, and the relatively short 12-month follow-up period. Future studies should evaluate long-term clinical outcomes of genotype-guided versus standard treatment approaches, conduct comprehensive cost-effectiveness analyses of routine genetic testing implementation, and assess tailored antiplatelet strategies optimized for different metabolizer phenotypes.

## CONCLUSION

The study revealed that, either or both functional alleles of CYP2C19 *2 (rs4244285) were lost in 57.84% patients of Khyber Pakhtunkhwa. Our results showed a strong association between GA and AA genotypes, classified as intermediate and poor metabolizers, respectively, and an increased incidence of cardiovascular events. While, patients with the GG genotype who were classified as normal metabolizers were significantly less likely to have cardiovascular events. These findings suggest that CYP2C19 *2 genotyping may serve as a valuable predictive marker for cardiovascular risk stratification and could potentially guide personalized therapeutic interventions.

### Authors Contributions:

**AR:** Designed and conducted the research, gathered, validated and analyzed the data, and developed the initial manuscript. Responsible and accountable for the integrity and accuracy of the entire work.

**SS:** Reviewed the manuscript and closely supervised and monitored all aspects of this study from conception of the idea to paper submission.

**STH:** Selected and sorted out the patients, supervised the research, conception of idea, critically revised and edited the manuscript. Responsible and accountable for the integrity and accuracy of the entire work.

**WI:** Sampling, data entry and data analysis

**MA:** Supervised lab work, analyzed the data and critically reviewed the manuscript.

## References

[1] Ibanez B, James S, Agewall S, Antunes MJ, Bucciarelli-Ducci C, Bueno H (2018). 2017 ESC Guidelines for the management of acute myocardial infarction in patients presenting with ST-segment elevation:The Task Force for the management of acute myocardial infarction in patients presenting with ST-segment elevation of the European Society of Cardiology (ESC). Eur Heart J..

[2] Zhu Y, Zhou J, Romero ELJB, Letters MC (2022). Reinvestigation of clopidogrel bioactivation unveils new cytochrome P450-catalyzed thioester cleavage mechanism. Bioorg Med Chem Lett..

[3] Chen YW, Liao YJ, Chang WC, Hsiao TH, Lin CH, Hsu CY (2022). CYP2C19 loss-of-function alleles predicts clinical outcomes in East Asian patients with acute myocardial infarction undergoing percutaneous coronary intervention and stenting receiving clopidogrel. Front Cardiovasc Med..

[4] Giantini A, Timan IS, Dharma R, Sukmawan R, Setiabudy R, Alwi I (2023). The role of clopidogrel resistance-related genetic and epigenetic factors in major adverse cardiovascular events among patients with acute coronary syndrome after percutaneous coronary intervention. Front Cardiovasc Med..

[5] Stone GW, Witzenbichler B, Weisz G, Rinaldi MJ, Neumann F-J, Metzger DC (2013). Platelet reactivity and clinical outcomes after coronary artery implantation of drug-eluting stents (ADAPT-DES):a prospective multicentre registry study. Lancet..

[6] Al-Rubaish AM, Al-Muhanna FA, Alshehri AM, Al-Mansori MA, Alali RA, Khalil RM (2021). Bedside testing of CYP2C19 vs. conventional clopidogrel treatment to guide antiplatelet therapy in ST-segment elevation myocardial infarction patients. Int J Cardiol..

[7] Samad Z, Hanif BJC (2023). Cardiovascular diseases in Pakistan:imagining a postpandemic, postconflict future.

[8] Svilaas T, Vlaar PJ, van der Horst IC, Diercks GF, de Smet BJ, van den Heuvel AF (2008). Thrombus aspiration during primary percutaneous coronary intervention. N Engl J Med..

[9] Ibánez B, James S, Agewall S, Antunes MJ, Bucciarelli-Ducci C, Bueno H (2017). 2017 ESC Guidelines for the management of acute myocardial infarction in patients presenting with ST-segment elevation. Europ Heart J..

[10] Nawaz U, Noor M, Waheed A (2023). Cytochrome P-450 CYP2C19 genetic polymorphism and its relation with clopidogrel resistance. J Pak Med Assoc..

[11] Ahmed S, Gul S, Siraj S, Hussain A, Sheikh FS, Shah SU (2022). Antiplatelet response to clopidogrel is associated with a haplotype in CYP2C19 gene in Pakistani patients. Sci Rep..

[12] Riaz S, Muhammad Din S, Usman Tareen M, Tariq F, Latif Y, Siddiqi S (2019). Genetic Polymorphism of CYP2C19 in Pakistani Population. Iran J Pharma Res..

[13] Sattar S, Ahmed N, Akhter Z, Aijaz S, Lakhani S, Malik R (2019). In-Hospital outcomes in acute coronary syndrome patients with concomitant severe chronic kidney disease undergoing percutaneous coronary intervention. Pak J Med Sci..

[14] Otowa K, Kohsaka S, Sawano M, Matsuura S, Chikata A, Maruyama M (2022). One-year outcome after percutaneous coronary intervention in nonagenarians:Insights from the J-PCI OUTCOME registry. Am Heart J..

[15] Griffioen AM, van den Oord SC, Teerenstra S, Damman P, van Royen N, van Geuns RJMJJotSfCA (2022). Clinical relevance of impaired physiological assessment after percutaneous coronary intervention:a meta-analysis. J Soc Cardiovasc Angiogr Interv..

[16] Riaz S, Din SM, Tareen MU, Tariq F, Latif Y, Siddiqi S (2019). Genetic polymorphism of CYP2C19 in Pakistani population. Iran J Pharm Res..

[17] Voicu V, Diehm N, Moarof I, Parejo S, Badiqué F, Burden A (2024). Antiplatelet therapy guided by CYP2C19 point-of-care pharmacogenetics plus multidimensional treatment decisions. Pharmacogenomics. Pharmacogenomics..

[18] Park JJ, Cabel GY, Cheng KK, Dang J, Ardati AK, Han J (2024). Genotype-guided prescribing predictors in CYP2C19 intermediate metabolizers receiving percutaneous coronary intervention. Pharmacogenomics..

